# Digital Circuit for Seamless Resampling ADC Output Streams†

**DOI:** 10.3390/s20061619

**Published:** 2020-03-14

**Authors:** Mauro D’Arco, Ettore Napoli, Efstratios Zacharelos

**Affiliations:** Department Electrical and Information Technologies Engineering (DIETI), University of Naples Federico II, via Claudio 21, 80125 Naples, Italy; ettore.napoli@unina.it (E.N.); efstratios.zacharelos@unina.it (E.Z.)

**Keywords:** resampling, interpolating polynomial, polyphase filter, digital circuit design, FPGA, ASIC

## Abstract

Fine resolution selection of the sample rate is not available in digital storage oscilloscopes (DSOs), so the user has to rely on offline processing to cope with such need. The paper first discusses digital signal processing based methods that allow changing the sampling rate by means of digital resampling approaches. Then, it proposes a digital circuit that, if included in the acquisition channel of a digital storage oscilloscope, between the internal analog-to-digital converter (ADC) and the acquisition memory, allows the user to select any sampling rate lower than the maximum one with fine resolution. The circuit relies both on the use of a short digital filter with dynamically generated coefficients and on a suitable memory management strategy. The output samples produced by the digital circuit are characterized by a sampling rate that can be incoherent with the clock frequency regulating the memory access. Both a field programmable gate array (FPGA) implementation and an application specific integrated circuit (ASIC) design of the proposed circuit are evaluated.

## 1. Introduction

In the majority of digital storage scopes (DSOs) the analog-to-digital converter (ADC) always works at its maximum sampling rate, imposed by an internal fixed frequency clock [1,2]. The user can also select lower sampling rates, which are achieved by seamlessly resampling the ADC output stream. Resampling is performed by means of a digital circuit that interfaces ADC and acquisition memory, and merely consists in decimating the input stream, which involves grouping the samples at the maximum sampling rate into consecutive sets, and acquiring, that is, storing in the acquisition memory, only the first sample of each set. All sets have the same size, which is equal to the required decimation factor—for instance, grouping samples into sets with size equal to 2 means acquiring one every other sample, thus halving the input sampling rate [3,4].

Resampling based on decimation is characterized by the following drawbacks: (i) the selection of the sampling rate is limited to the values that can be obtained dividing the maximum sampling rate by integer values; (ii) if the selected sampling rate is less than the Nyquist rate of the analog input, the acquired signal is corrupted by aliasing [5,6].

In general, fine selection of the sample rate improves the performance of the DSO, allowing more efficient usage of memory resources. In fact, a limited set of sample rates implies a limited set of time windows for signal observation. Due to these limitations, it is possible that the analysis is performed observing the signal of interest in a time window where up to almost 50% of the window contains useless samples. Many DSOs are also complemented with math capabilities like Fast Fourier Transform (FFT) options that allow frequency domain analyses. In these applications the choice of the sample rate determines, in conjunction with the memory size, the frequency span and resolution settings; the limitations characterizing the sample rate selection lead to sub-optimal settings. Some DSOs allow the user applying an external clock signal to control the sampling rate. This option is not very common because of the following drawbacks: (i) the external path has a limited bandwidth, much inferior to that of the internal path, so that the operative range of the DSO is substantially reduced; (ii) some functionalities of the instrument, which cannot work with the external clock, are disabled; (iii) the precision specifications of the DSO, which are related to the operation with the internal clock, cannot be used to evaluate the accuracy of the measurement results.

In theory, fine control of the sampling rate in real-time DSOs can be obtained by resampling the ADC output stream by means of more effective methods alternative to hard decimation [7,8,9]. These methods can be inherited by digital signal processing theory, and rely either on the use of interpolation algorithms or polyphase filters [10,11]. The first method allows varying the sampling rate dynamically, and puts no restrictions on the selection of the output sampling rate. The second method is instead limited to decimation factors that are equal to $\frac{L}{M}$, where *L* and *M* are integers. Both methods counteract aliasing effects by means of low-pass filtering operations, which are implicit in the interpolation algorithm, and explicit in the processing scheme of polyphase filters [12,13,14,15]. In fact, the use of an interpolation function is equivalent to filtering the signal with a filter characterized by a frequency response where the number of taps is equal to the number of points used in interpolation. Unfortunately, the hardware implementation of both methods is difficult due to the strict requirements of seamless operation and fine resolution in sampling rate selection [16,17,18].

A method that shows a viable solution to finely control the sampling rate in DSOs has been presented in Reference [19], and a digital circuit that implements this method using field programmable gate array (FPGA) technology has been illustrated at the ApplePies 2019 Conference [20]. In detail, the digital circuit exploits a resampling method based on linear interpolation, which trades-off between accuracy and circuit complexity. It is designed to work between the ADC and the acquisition memory, and allows selecting sampling rates from the highest frequency, $f_{ck}$, down to its half value, $\frac{f_{ck}}{2}$. Choosing a sample rate lower than $\frac{f_{ck}}{2}$ is easily obtained by cascading the proposed circuit with a standard one that performs decimation by an integer value. The acquisition chain is made up of ADC, proposed digital circuit, and acquisition memory, all operating synchronously at the system clock rate $f_{ck}$. It provides samples that represent a version of the input signal characterized by a sample rate $f_{s}=Cf_{ck}$, where *C* is a fractional value in the interval ($\frac{1}{2}$, 1). The defining resolution of *C* is only limited by the number of bits adopted in its binary representation; the reciprocal of *C* can be regarded as a non-integer decimation factor.

This work is an extended version of the article published in the Conference Proceedings [20]. It takes into consideration several different methods for DSOs sampling rate control, and, by evaluating their performance highlights how the proposed digital circuit represents a good compromise between achievable accuracy and circuit complexity. Starting from the primary version of the circuit, an improved version characterized by different pipeline levels is developed, and an application specific integrated circuit (ASIC) design of the proposed solution is also analyzed [21,22,23].

The paper discusses more about the resampling methods based on interpolation and polyphase filters in Section 2. The performance of different interpolators, which satisfy the requirements of effective hardware implementation and high resolution in sampling rate selection, is analyzed in Section 3 through simulations. Section 4 illustrates the design of the proposed digital circuit, and, finally, Section 5 gives concluding remarks.

## 2. Methods

In general, resampling a mono-dimensional signal, defined upon a sampling grid, aims at producing another representation of the same signal, referred to a different sampling grid. It basically requires gaining the samples referred to the output grid by processing the available ones. Resamplers manage a redundant representation of the signal, that includes both the input samples and the resampled ones; the second are the only ones returned by the circuit.

In the most common resampling applications both the input and output sampling grid are uniform and the circuit has to deal with samples that are streamed at regular time instants, such that a sampling rate is defined. Also, resampling has to be performed real-time seamlessly on the input stream, which is very challenging, especially in the presence of high-rate data streams [24,25,26,27].

Hereinafter, the attention is mainly paid to real-time seamless resampling of signals that are naturally defined in the time domain, for which the input sampling rate needs to be changed into a different sampling rate, lower than the input one. The methods that are illustrated can be adapted to other signals defined in different domains by exploiting the unique correspondence between the points of the sampling grids and the related time-stamps in their streamed form produced at the processing stage [28,29].

### 2.1. Resampling Based on the Use of Approximating Polynomials

The most straightforward resampling approach, capable of granting real-time seamless performance, exploits the zero-order interpolation process, which assumes the signal constant until the next sample is available. In other terms, the resampled value, x(n+t), where *t* is a fraction of the sampling period, $T_{s}$, (reciprocal of the sampling rate, $f_{s}$) of the input stream, is assumed equal to that of the most recent sample x(n) [30,31].

Alternatively, the first-order or linear interpolator can be used to improve the accuracy of the resampling process. Linear interpolators wait for the subsequent sample of the input stream x(n+1) to compute the value of any sample at a time instant in the midst. Specifically, they compute it by adding to x(n) a term equal to *t* times the time derivative, which is estimated as first forward difference [32,33].

More generally, resampling can rely on interpolators that use a larger set of samples adjacent to the resampling instant to determine the resampled value. The samples of the set are processed to identify a polynomial of the *t* variable, P(t), that locally approximates the signal behavior. The value of the polynomial at the resampling instant provides the resampled value. The polynomial is identified imposing constraints that can involve the values of the signal and/or of its time derivatives. The most common solutions are:

- the approximating polynomial connects all the samples of the set (Lagrange polynomial) and is characterized by a degree equal to the number of samples of the set minus 1;
- the approximating polynomial is identified by fitting the samples in order to minimize the mean square error, and is characterized by a degree less than the number of samples of the set minus 1;
- the approximating polynomial connects a subset of the samples and has the same time derivative of the signal in those points (Hermite polynomial).

In all the aforementioned approaches the resampled value obtained using an approximating polynomial can be represented with a matrix formulation. For instance, for a 3-degree approximating polynomial, one as:

(1) $$\begin{array}{ccc} x(n+t) & = & c_{1}\left(n\right)+c_{2}\left(n\right)t+c_{3}\left(n\right)t^{2}+c_{4}\left(n\right)t^{3}=\\ & = & \left[\begin{array}{cccc} 1 & t & t^{2} & t^{3} \end{array}\right]\left[\begin{array}{cccc} a_{11} & a_{12} & a_{13} & a_{14}\\ a_{21} & a_{22} & a_{23} & a_{24}\\ a_{31} & a_{32} & a_{33} & a_{34}\\ a_{41} & a_{42} & a_{43} & a_{44} \end{array}\right]\left[\begin{array}{c} x_{n-1}\\ x_{n}\\ x_{n+1}\\ x_{n+2} \end{array}\right] \end{array}$$

where x(n+t) is the resampled value at time instant n+t, *t* is within the interval (0,1), and each coefficient, $a_{ij}$, *i* = 1, …, 4, is a linear combination of the values of the 4 consecutive samples $\left\{x_{n-1},x_{n},x_{n+1},x_{n+2}\right\}$ with constant coefficients, namely:

(2) $$c_{i}\left(n\right)=\sum_{j=1}^{4}a_{ij}x_{n-2+j}.$$

The constant coefficients $a_{ij}$ can be determined imposing the constraints used to define the approximating polynomial. Hence, for a Lagrange polynomial, one can consider the system of equations obtained imposing that the polynomial connects the values $\left\{x_{n-1},x_{n},x_{n+1},x_{n+2}\right\}$ characterized, respectively, by *t* abscissas $\left\{-1,0,1,2\right\}$:

(3) 1−1+1−1100011111248c1c2c3c4=xn−1xnxn+1xn+2

from which the $a_{ij}$ values are determined by inverting the coefficient matrix in (3) as:

(4) aij=inv1−1+1−1100011111248=160600−2−36−13−630−13−31

If an approximating polynomial of second degree is selected, then only three coefficients, $c_{i}\left(n\right),i=1,2,3$, that are still linear combination of the samples $\left\{x_{n-1},x_{n},x_{n+1},x_{n+2}\right\}$ with constant coefficients are needed. These coefficients can be determined imposing the same constraints adopted to identify the Lagrange polynomial, i.e:

(5) 1−1+1100111124c1c2c3=xn−1xnxn+1xn+2

but, since a second degree polynomial cannot in general grant the connection of more than 3 points, one has to accept an approximate solution that best fits the data according to a given cost function. The solution that grants the least mean square error, as well known, is obtained solving the system in (5) using the pseudo-inverse matrix method; in this case the $a_{ij}$ values, *i* = 1, …, 3, *j* = 1, …, 4, are:

(6) aij=inv1+1+1+1−101210141−1+11001111241111−10121014==1203119−3−113715−5−55

The coefficients of a 3-degree Hermite polynomial are identified using also the time derivative of the approximating polynomial P(t), namely:

(7) $$\frac{dP}{dt}=c_{2}\left(n\right)+c_{3}\left(n\right)t+c_{4}\left(n\right)t^{2}$$

to form a system of equations that imposes that the polynomial connects the central samples, referred to the *t* abscissas equal to 0 and 1, and has the same derivative of the signal in those points. In matrix form, these constraints can be expressed by:

(8) 1000111101000123c1c2c3c4=1202+0+00020−10100−101xn−1xnxn+1xn+2

where the time derivative of the signal is estimated in terms of finite central difference. The $a_{ij}$ values, i,j = 1, …, 4, are obtained solving system (8) as:

(9) aij=12inv100011110100012302+0+00020−10100−101=120200−10102−54−1−13−31

The equations that define the interpolation methods discussed above can be summarized as in Table 1.

### 2.2. Resampling Based on Polyphase Filters

Resampling with polyphase filters is commonly performed in a variety of systems, like multipurpose receivers, where several different sampling rates are supported to process signals characterized by different bandwidths, as well as in digital audio and video systems, and so forth [34,35]. In these systems the signal is initially sampled at a high sampling rate, then processed to modify the sampling rate by a factor $\frac{L}{M}$. Processing involves interpolation by *L*, low-pass filtering, and decimation by *M*. Low-pass filtering removes the image frequencies due to sampling rate changes; it is implemented using polyphase decomposition of both the input signal and filter coefficients [36,37].

**Figure 1:**
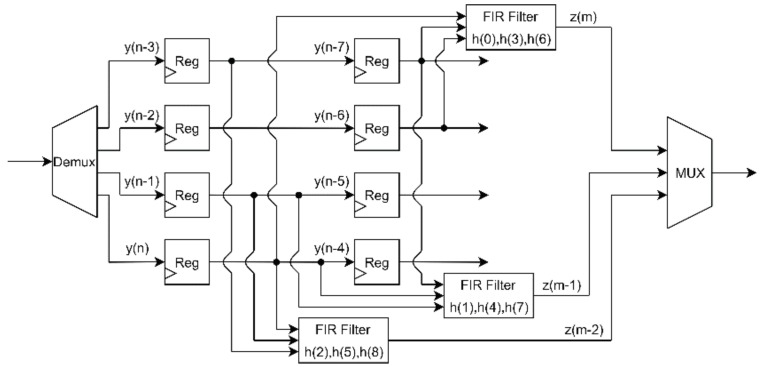
Schematic of a digital resampler implementation based on polyphase decomposition. Resampling factor equal to 3/4 low-pass filter with 9 taps.

For the sake of clarity, an example of a $\frac{3}{4}$-resampler that uses a short low pass filter with 9 coefficients, h(n) = $\left\{h(0),h(1),\ldots h(8)\right\}$, is shown in Figure 1. The input signal y(n) is de-multiplexed in order to retrieve 4 consecutive samples and route them to 4 individual channels with a single operation. The output of the resampler, z(m), is obtained by multiplexing the outputs produced by 3 filters, each filter defined in terms of 3 coefficients of h(n) according to polyphase decomposition rules.

Polyphase filters are characterized by low requirements in terms of clock frequency and can be set to both up-sample and down-sample the input stream, but are not suitable for programmable resampling factors, because any polyphase structure is defined by the same ratio between the input and output sampling rate; consequently, any change of the resampling ratio implies modifying their structure [38].

### 2.3. Pro and Cons of Approximating Polynomials and Polyphase Filters

Resampling with polyphase filters straightforwardly changes the input sampling rate, $f_{ck}$ to the output one $f_{s}=\frac{L}{M}f_{ck}$. In fact, thanks to the use of a demultiplexer at the front-end, the polyphase filter processes any $\frac{T_{ck}}{M}$ seconds ($T_{ck}=f_{ck}^{-1}$) a set of *M* input samples and returns a set of *L* output samples, which are written in the acquisition memory with a single memory access, thus lowering the input sampling rate by a factor $\frac{L}{M}$. Unfortunately, any change of the sampling rate requires re-programming the digital circuit. Although, in theory, re-programming can be done, in case of sampling rates that involve very large *M* and *L* values, one should reserve sufficient hardware resources for huge polyphase structures, seldom required and largely unused; nonetheless, the responsiveness of the system would definitely slow-down.

Resamplers based on interpolators are instead less demanding in terms of hardware resources and allow controlling the sampling rate easily. They also require a suitable strategy for arranging the lower sampling rate output stream. Specifically, the digital resampler can take as input both a set of consecutive samples and the *t* variable, as specified by the interpolation equations summarized in Table 1. It can run at a clock rate equal to the input sampling rate, quantifying the *t* variable as the delay of the resampling instant with respect to the discrete time *n*. To this end, it can exploit an accumulator that increments by $\frac{T_{s}}{T_{ck}}-1$ ($T_{s}=f_{s}^{-1}$) any $T_{ck}$ seconds. The accumulator represents the *t* variable except when it overflows a unitary value. The overflow repeatedly occurs with a cadence related to the selected sampling rate. Overflow means that the resampling instant does not fall between the discrete time n−1 and *n*, but is in the midst of *n* and n+1, such that it should be considered at the next processing step. At any occurrence of an overflow, the digital circuit skips the calculation of the resampled value, and performs a unitary decrement of the accumulator at the subsequent clock cycle, thus restoring *t* between the expected discrete time instants.

The use of approximating polynomials is preferred in the development of a digital circuit aimed at granting fine control of DSOs sampling rate, because it has several interesting features. These include the capability of resampling even if the ratio of sample rates is not rational, as well as of seamlessly managing real-time streams even in the presence of time-varying sample rates.

## 3. Simulation Analyses

As well known, changing the sampling rate produces aliasing, which is usually counteracted by filtering the digital signal with a low pass filter before resampling. The performance of the resampling methods is affected by the presence of residual alias, thus the frequency response of the adopted anti-aliasing filter must be taken into account. The anti-aliasing filter is implicit in the resampling approach based on the use of an approximating polynomial, and its impulse response is given, in general, by a set of coefficients that depend on *t*; for instance, from Equation (1), one can obtain the coefficients of the 4-tap filter, $d_{j},j=1,\ldots ,4$ as:

(10) $$d_{j}=\sum_{i=1}^{4}a_{ij}t^{j-1}.$$

**Figure 2:**
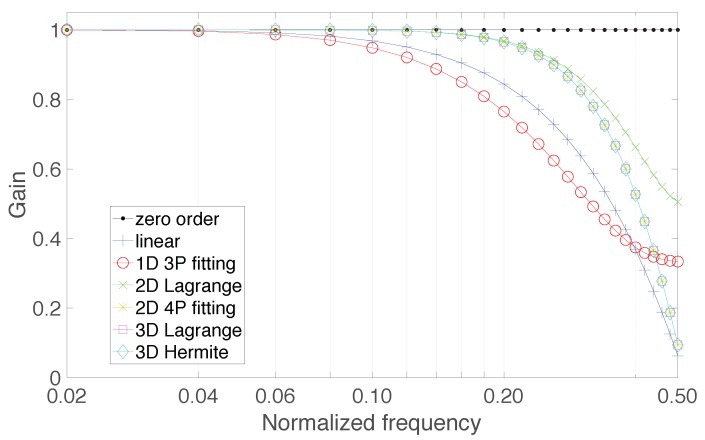
Frequency response of the anti-alias filters implicit in the resampling approaches based on the use of approximating polynomials.

An estimation of the residual alias can be approached taking into account that the spectrum of a digital signal is periodic with unitary period, and that lowering the sampling rate down to $f_{s}=Cf_{ck}$ has the effect of replicating the spectrum at a pace equal to *C*. Moreover, since the *t* variable changes during the resampling process, ranging in the interval (0, 1), the features of the anti-aliasing filters change as well. Anyway, taking into account that *t* is within (0, 1) and, on average, $t=\frac{1}{2}$, one determines the average behavior of the filter. Using the frequency response, H(ν), of the filter that describes the average behavior, which is gained by taking the Fourier transform of the filter coefficients estimated with $t=\frac{1}{2}$, allows representing the spectrum of the resampled version as:

(11) $$Z\left(\nu \right)=\sum_{p,q=-\infty }^{\infty }H\left(\frac{\nu }{C}\right)X\left(\frac{\nu -p}{C}-q\right),$$

where ν is normalized to the sampling rate $f_{ck}$. From Equation (11) the alias-free version of the resampled signal can be obtained using p=q=0, whereas all the combinations satisfying $\left|p-Cq\right|<\frac{C}{2}$ identify the residual aliases that fall in the spectrum of the resampled signal. Figure 2 shows the frequency response of the anti-alias filters that are implicit in the 7 approaches detailed in Table 1. The different responses are characterized by specific markers and colors: ’plus’ marker and blue color is for the linear interpolator, ’circle’ marker and red color for the first-degree polynomial fitting 3 sample points, ’x’ marker and green color for the second-degree Lagrange polynomial, ’star’ marker and yellow color for the second-degree polynomial fitting 4 sample points, ’square’ marker and magenta color for the third-degree Lagrange polynomial, and, finally, ’diamond’ marker and cyan color for the third-degree Hermite polynomial (a suitable legend has been included to highlight these correspondences). For the sake of completeness also an additional graphic, related to the zero-order resampling approach, is shown using ’dot’ marker and black color to highlight the all-pass nature of this approach, which is detrimental because it provides no mitigation of aliasing effects.

The frequency responses given in Figure 2 are obtained by Fourier transforming the impulse response estimated upon 50 points, and consist of 25 bins, equally spaced at a pace of 0.02; they show the behavior of the filters up to the normalized frequency 0.5, corresponding to $\frac{f_{ck}}{2}$ hertz. One can observe that the approximating polynomials with higher degree offer flatter gain and better selectivity. Also, the mean behavior of the anti-aliasing filters related to the use of the second-degree polynomial fitting 4 sample points, the third-degree Lagrange polynomial, and the third-degree Hermite polynomial are identical. The ideal frequency response behavior should exhibit unitary gain in the interval $\left(0,\frac{C}{2}\right)$, to avoid undesired attenuation of the signal spectral content, and zero gain in $\left(\frac{C}{2},\frac{1}{2}\right)$ to cancel any alias contribution.

**Figure 3:**
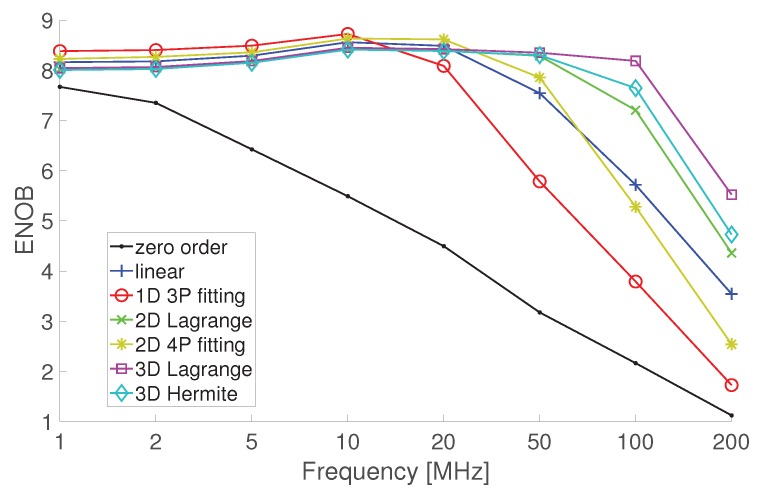
Effective number of bits (ENOB) offered by the resampling approaches based on the use of approximating polynomials.

Although the anti-aliasing filter plays an important role in the resampling process, the analysis of its mean behavior provides only partial insight, since the time-varying nature can play a role that cannot be analyzed using Equation (11). A deep insight in the performance of the resampling methods can instead be gained by using the standard test methods for ADC, detailed in Reference [39], such as the effective number of bits (ENOB) and the spurious-free dynamic range (SFDR). The first is a measure of the signal-to-noise and distortion ratio used to compare the actual ADC performance to an ideal one; the latter considers, in the presence of a pure sine-wave input, the ratio of the amplitude of the output spectral component at the input frequency, $f_{0}$, to the amplitude of the largest harmonic or spurious spectral component. Figure 3 shows the ENOB offered by the considered methods in the presence of test sine-waves.

The simulations have considered samples quantized by an 8 bit ADC. Quantization has been applied to a signal corrupted by white Gaussian noise, with rms value equal to 15% of the LSB of the ADC. The sampling rate of the input stream is $f_{ck}$ = 1 GSa/s, that is resampled at $f_{s}$ = 743 MHz, thus *C* = 0.743. The sine-waves adopted in the tests are characterized by the frequency values $\left\{1,2,5,10,20,50,100,200\right\}$ MHz. The results show that ENOB obtained after resampling can even improve in the presence of the lower input frequencies of the considered set with respect to the nominal 8 bit. This is due to the anti-aliasing low-pass filter that reduces the acquired bandwidth and thus also the distortion due to quantization. As the input frequency approaches the upper limit of the Nyquist bandwidth, the performance of all methods rapidly decreases, and one can observe that the methods that use approximating polynomials with higher degree can grant ENOB close to the nominal number of bits on wider ranges. As expected, the effectiveness of interpolation algorithms diminishes as soon as the input sinusoidal signal is sampled collecting a few points per period, namely 7–8 points. This happens because the algorithms consider the local behavior of the signal, whereas the uniform sampling theorem claims for interpolation with sinc functions that consider the behavior on the whole time axis; unfortunately sinc interpolation is unfeasible and its straightforward approximations, like those based on the use of truncated sinc functions, are characterized by huge computational burden, which is not compatible with real-time execution. As a rule of thumb, suggesting some oversampling in the use of the acquisition mode with fine selection of the sample rate, avoids incurring in poor results.

**Figure 4:**
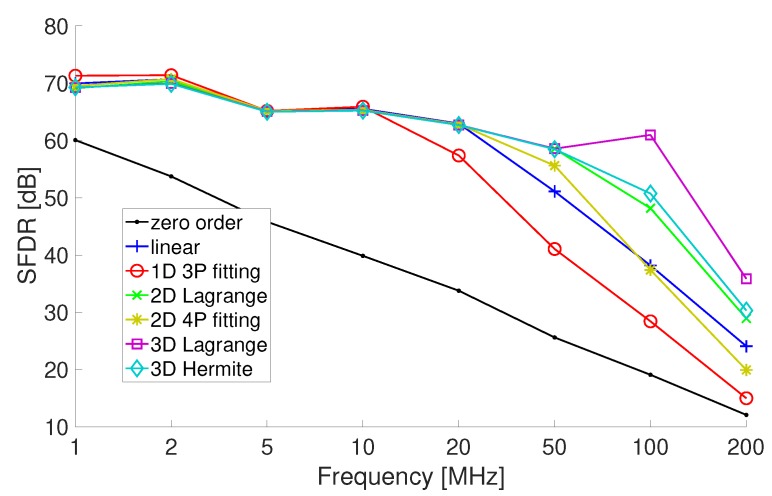
Spurious-free dynamic range (SFDR) offered by the resampling approaches based on the use of approximating polynomials.

The simulations have also estimated for the same test set-up the SFDR in order to highlight if the time-varying behavior of the anti-alias filters introduce relevant spurious; the obtained results are shown in Figure 4.

The performance parameters highlight the convenience of using Lagrange or Hermite polynomials (the linear interpolation coincides with the adoption of a first-degree Lagrange polynomial) for interpolation rather then zero-order or fitting polynomials based methods.

**Figure 5:**
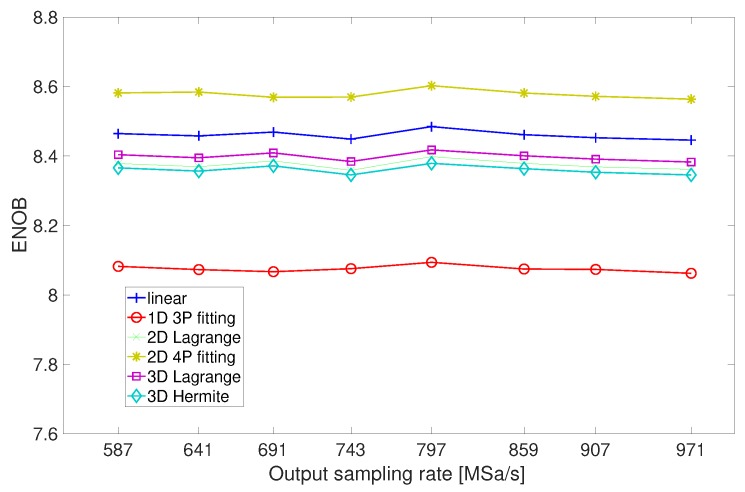
ENOB offered by the resampling approaches in the presence of an input sine-wave at 20 MHz when the 1 GHz input sampling rate is lowered down to frequencies of the set {587,641,743,797,859,907,971} MSa/s.

Further simulations have been addressed to the analysis of any dependence of the performance on the output sampling rate. As an example, Figure 5 shows the ENOB obtained in the presence of a sine-wave input at 20 MHz when the 1 GHz input sampling rate is lowered down to the frequencies of the set {587,641,743,797,859,907,971} MSa/s.

The simulations highlight that the performance is unaffected by sampling rate changes; all the methods offer ENOB constant and above 8 bits, except for the zero-order method, the performance of which, although independent of the output sampling rate, is largely below the lower axis limit utilized in Figure 5.

## 4. Proposed Digital Circuit

### 4.1. Operation Details

The proposed digital circuit implements the linear interpolation method that represents a good compromise between accuracy and circuit complexity. It processes in real-time the signal x(n) streaming out of the ADC, and returns the output, y(n); both are characterized by the clock rate, $f_{ck}$, but y(n) contains a resampled version of x(n) characterized by a sampling rate $f_{s}=Cf_{ck}$.

More specifically, the value y(n) is determined by combining the samples x(n−1) and x(n) returned by the ADC according to:

(12) $$\begin{array}{ccc} y(n) & = & a(n)x(n-1)+(1-a(n))x(n)=\\ & = & a(n)x(n-1)+b(n)x(n) \end{array}$$

where a(n) is a time-varying coefficient, updated at every clock cycle by subtracting to its current value a quantity, chosen by the user, and related to the sampling factor as $\frac{1-C}{C}$. Notice that the aforementioned variable *t* corresponds to the variable b(n) of the Equation (12), and consequently a(n)=1−t. Subtraction is skipped if the current value of the coefficient a(n) is negative, and in its place an addition by one is performed. Hence, the output of the digital circuit y(n) contains, with some redundancy, the resampled version of x(n).

The circuit also produces a signal $Ptr_{X}$, that indicates the memory location where y(n) is stored. The generated sequence y(n) is stored in memory at system frequency, $f_{ck}$ but, in order to cope with the lower sampling rate, $Ptr_{X}$ is not incremented when the a(n) coefficient is incremented by one. In this way, two consecutive outputs share the same value of $Ptr_{X}$, which means that the second one overwrites the first.

**Figure 6:**
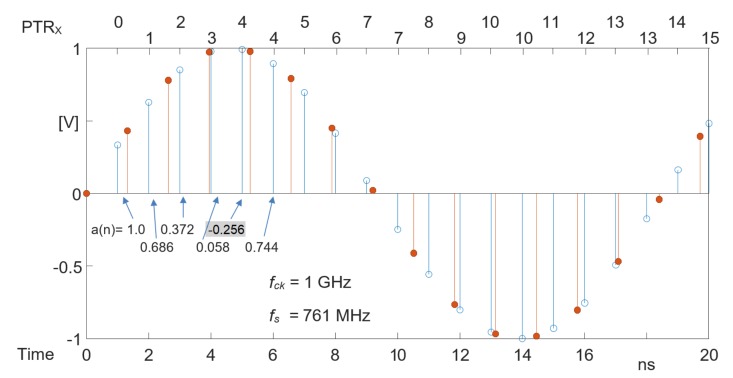
Example sequences for a(n) and $Ptr_{X}$.

An example will better clarify the meaning of a(n). In Figure 6 a sinusoidal signal at 54 MHz is shown. It is sampled with the 1 GHz ($T_{ck}$ = 1.0 ns) system clock (sampling shown with circles). The result obtained resampling at 761 MHz ($T_{s}$ = 1.314 ns) is shown with red bullets. The resampling factor is *C* = 0.761, and the coefficient a(n) is updated subtracting $\frac{1-C}{C}$ = 0.3141 to the current value. Variable b(n) represents the point inside the sampling period where resampling must be performed. The bottom axis is the time while the top axis shows the increment of the memory pointer. When a(n) is incremented (time: 6, 10, 14, 18 in Figure 6) the memory pointer is not updated.

### 4.2. Design Details

**Figure 7:**
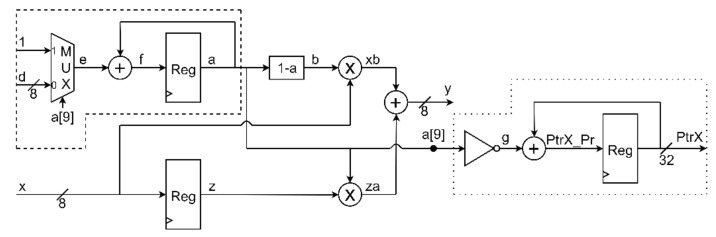
Circuital implementation of the proposed algorithm.

**Figure 8:**
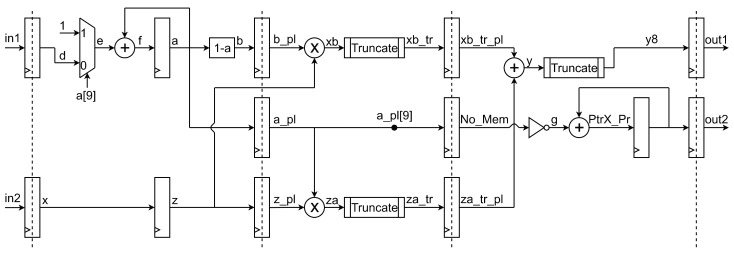
Circuital implementation of the proposed algorithm with pipeline registers (pipeline levels are highlighted with dashed lines).

A digital circuit for the implementation of the proposed resampling algorithm has been designed. The schematics before and after pipelining are in Figure 7 and Figure 8.

Circuit input data are the signal to be resampled *x*, and factor *d* = $\frac{C-1}{C}$. The output data are the resampled stream *y*, and the memory pointer, $Ptr_{X}$. The number of bits for *x*, *d*, and *y*, is 8, while the memory pointer, $Ptr_{X}$ is represented with 32 bits.

The two complementary coefficients, *a* and *b*, are multiplied by the previous value (*z* signal in Figure 7) and the current value of the input signal (*x* signal in Figure 7), respectively. Afterwards, the two products are summed, in order to produce the output signal, *y*, as indicated in Equation (12).

The updating of the coefficient *a*, relies on adding either the quantity *d*, or in the case of exception, a unitary value to the current value of *a*. In the case of exception, *a* is negative, and the most significant bit (MSB) of the coefficient, is high, $a\left[9\right]$=1; otherwise, $a\left[9\right]$ = 0, and *d* is added to the current value of *a*. This distinction is realized with the use of a multiplexer, controlled by the MSB of signal *a*. After the correct choice between “1” and “*d*”, an accumulator is implemented for the updating of *a*.

A second accumulator is implemented, for the memory management. When *a* is positive, $a\left[9\right]$=0, *g*=1, and $Ptr_{X}$ is incremented by a unitary value. In the case of exception, *a* is negative, $a\left[9\right]$=1, *g*=0, and $Ptr_{X}$ remains unchanged. The above described memory management strategy allows to store only the resampled values. The fact that occasionally the memory pointer is not incremented reflects the fact that after resampling the number of samples is less than that of the input signal.

In Figure 8 the pipelined resampler can be observed. The four vertical dashed lines mark the four pipeline levels introduced to the circuit in order to isolate the combinational logic, thereby achieving a lower clock period and a higher throughput. On the other hand, latency and chip area are increased. The number of flip-flops (registers) used for the pipeline implementation is: (8 + 8) + (10 + 10 + 8) + (12 + 1 + 12) + (8 + 32) = 109.

### 4.3. Implementation and Performance

As mentioned earlier, 8 bits are used for the representation of *d*, where *d* is within (−1, 0). However, during the experimental procedure, a 16-bit signal was also tried out in order to test the performance of the circuit. For an *n*-bit signal, the resolution obtained for *d* is constant and is equal to $2^{-n}$. The resolution obtained for *C* can be derived from:

(13) $$d\left(C\right)=\frac{d(C)}{d(d)}d\left(d\right)=\frac{d}{d(d)}\left(\frac{1}{1-d}\right)2^{-n}.$$

Given the fact that the relationship between *C* and *d* is not linear, the resulting resolution of *C* (the actual resampling factor) differs for different *d* values. In Table 2, some information related to the resolution of the resampling factor are presented. Assuming a 1 GHz clock frequency, using 8-bit for the *d* signal allows a frequency resolution that ranges from 390 kHz to 97.5 kHz, while using 16 bit the frequency resolution can be as low as 4 kHz.

The circuit is described in hardware description language (HDL) and a first assessment of the performance has been conducted with a high-end FPGA as a target. This aims to demonstrate the available performances in a reconfigurable environment. In Table 3, some basic features and resources are presented for the implementation on a StratixIV GX FPGA device by Altera. The implemented design is the one depicted in Figure 8 with *d* signal represented by 8 bits.

Tests similar to those considered in the simulation analyses have been repeated on sinusoidal signals, demonstrating that when the sampling frequency is at least ten times higher than the signal bandwidth, the results are satisfactory. For instance, in the presence of an input signal corrupted by white Gaussian noise (rms value equal to 15% the LSB of the ADC) and quantized by an 8 bit ADC, resampling at 743 MHz a 47.1 MHz signal converted with a 1GSs ADC has lowered the ENOB from 7.8 to 7.5 and left unaltered the SFDR, which is a quite limited degradation. The results do not exhibit recognizable changes if 50 kHz random deviations of the input frequency are considered.

An ASIC implementation has also been carried out. The circuit is synthesized by targeting a commercial standard-cell library in 14 nm fin field effect transistor (FinFET), from Global Foundry. Physical synthesis is performed by using Cadence Genus; no special cells are designed for the implementation and the circuit is automatically synthesized according to timing constraints. The considered technology corner is the typical one with 0.8 V of supply voltage and regular threshold voltage. The simulations, with delay and switching activity annotation, have been conducted with a suite of tools for the design and verification of ASICs and FPGAs, commonly referred to by the name NCSIM in reference to the core simulation engine. Power dissipation is computed by simulating the final netlist with 10,000 input vectors from an asynchronously sampled sinusoid to obtain the switching activity of each node.

While aiming for the highest frequency possible, several syntheses took place. Firstly, the circuit in Figure 8 was synthesized. Later the same circuit was synthesized, taking into account a retiming algorithm that moves the structural location of registers in order to improve the performance, while preserving the functional behavior at the outputs. Afterwards, two and three extra levels of pipeline were added to the design of Figure 8, and the synthesis was carried out with the retiming algorithm. The same syntheses were done for both an 8-bit and a 16-bit *d* signal and the results are reported in Table 4, and Table 5, respectively. As expected, the maximum working frequency is largely increased with respect to the FPGA implementation. Moreover, there is a trade-off between maximum frequency and chip area as well as power consumption.

A comparison between the FPGA implementation (Table 3) and the ASIC design (Table 4 and Table 5) in this particular application allows the following considerations. The FPGA design is composed by quite large blocks and uses the digital signal processing (DSP) blocks to efficiently perform the binary multiplication. This is very useful for the FPGA that can reach a remarkable speed for a reconfigurable target but leaves very little space for the arithmetic optimization and for the introduction of pipeline levels (e.g., a pipeline is not possible inside the DSP blocks). On the other hand, the ASIC design exploits a standard cell library with very small granularity and can choose among various design techniques for the arithmetic blocks. Also, the pipeline level can be moved freely inside the arithmetic block if needed. As a consequence, retiming and pipelining allow a large leap in circuit clock frequency (from 3.03 GHz to 5.26 GHz in the d = 8 bit case and from 2.70 GHz to 5.00 GHz in the d = 16 bit case).

Implementation results show that the circuit is able to reach the 5 GHz target in both cases. The effect of the retiming and the presence of the additional pipeline levels is seen in an increase of both area (mainly due to the additional Flip Flops) and power dissipation.

## 5. Conclusions

The paper has reviewed the main digital signal processing based methods for controlling the sampling rate in DSOs by means of digital resampling approaches. A digital circuit that offers a promising solution to grant more control of the sampling rate, with respect to the existing approaches, has then been discussed. The circuit can be deployed in the acquisition channel of any DSO to interface the internal ADC and the acquisition memory. It has been implemented on FPGA and evaluated. Also, the performance of an ASIC design of the same circuit has been investigated. The proposed solution can be exploited to effectively improve the sampling rate selection capability of DSOs, especially when the instrument does not permit the use of an external clock to drive the internal ADC.

## Figures and Tables

**Table 1:** Lagrange, Hermite and best fitting polynomial (in the sense of least square error) adopted in resampling.

Samples	Interpolation	Equation
1	zero order	x(n+t)=x(n)
2	linear	x(n+t)=x(n)+[x(n+1)−x(n)]t
3	linear (best fitting)	$x\left(n+t\right)=\frac{1}{3}\left[x\left(n-1\right)+x\left(n\right)+x\left(n+1\right)\right]+\frac{1}{2}\left[x\left(n+1\right)-x\left(n-1\right)\right]t$
3	quadratic (Lagrange)	$\begin{array}{ll} x\left(n+t\right) & =x\left(n\right)+\frac{1}{2}\left[x\left(n+1\right)-x\left(n-1\right)\right]t+\\ & +\frac{1}{2}\left[x\left(n-1\right)-2x\left(n\right)+x\left(n+1\right)\right]t^{2} \end{array}$
4	quadratic (best fitting)	$\begin{array}{ll} x\left(n+t\right) & =\frac{1}{20}\left[3x\left(n-1\right)+11x\left(n\right)+9x\left(n+1\right)-3x\left(n+2\right)\right]+\\ & +\frac{1}{20}\left[-11x\left(n-1\right)+3x\left(n\right)+7x\left(n+1\right)+x\left(n+2\right)\right]t+\\ & +\frac{1}{4}\left[x\left(n-1\right)-x\left(n\right)-x\left(n+1\right)+x\left(n+2\right)\right]t^{2} \end{array}$
4	cubic (Lagrange)	$\begin{array}{ll} x\left(n+t\right) & =x\left(n\right)+\frac{1}{6}\left[-2x\left(n-1\right)-3x\left(n\right)+6x\left(n+1\right)-x\left(n+2\right)\right]t+\\ & +\frac{1}{2}\left[x\left(n-1\right)-2x\left(n\right)+x\left(n+1\right)\right]t^{2}+\\ & +\frac{1}{2}\left[-x\left(n-1\right)+3x\left(n\right)-3x\left(n+1\right)+x\left(n+2\right)\right]t^{3} \end{array}$
4	cubic (Hermite)	$\begin{array}{ll} x\left(n+t\right) & =x\left(n\right)+\frac{1}{2}\left[x\left(n+1\right)-x\left(n-1\right)\right]t+\\ & +\frac{1}{2}\left[2x\left(n-1\right)-52x\left(n\right)+4x\left(n+1\right)-x\left(n+2\right)\right]t^{2}+\\ & +\frac{1}{2}\left[-x\left(n-1\right)+3x\left(n\right)-3x\left(n+1\right)+x\left(n+2\right)\right]t^{3} \end{array}$

**Table 2:** Resampling factor resolution.

Name	Value
Best C-Step (8 bits)	$9.76\cdot 10^{-4}\left(C=0.500976\right)$
Worst C-Step (8 bits)	$39\cdot 10^{-4}\left(C=0.996094\right)$
Best C-Step (16 bits)	$0.04\cdot 10^{-4}\left(C=0.500004\right)$
Worst C-Step (16 bits)	$0.15\cdot 10^{-4}\left(C=0.999985\right)$

**Table 3:** Basic Features of the Resampler and FPGA resources (StratixIV-EP4SGX230KF40 implementation).

Name	Value
Maximum Clock Frequency	400 MHz
Combinational ALUTs	532 (<1%)
Dedicated Logic Registers	147 (<1%)
DSP Block 18-bit Elements	3 (<1%)

**Table 4:** ASIC implementation results for the resampler in 14 nm FinFET from Global Foundry technology using 8 bits for *d* signal.

	Clock Frequency [GHz]	Cell Count	Total Area $\left[\mu m\right]$	Flip Flops	Dynamic Power $\left[\frac{\mu W}{\mathrm{MHz}}\right]$	Leakage Power [μW]
basic	3.03	1052	584	145	1.190	0.707
retimed	3.85	1035	620	239	1.184	0.568
2 extra PL	5.26	1092	683	307	1.392	0.665
3 extra PL	4.55	1066	712	341	1.524	0.693

**Table 5:** ASIC implementation results for the resampler in 14 nm FinFET GF technology using 16 bits for *d* signal.

	Clock Frequency [GHz]	Cell Count	Total Area $\left[\mu m\right]$	Flip Flops	Dynamic Power $\left[\frac{\mu W}{\mathrm{MHz}}\right]$	Leakage Power [μW]
basic	2.70	1780	916	179	1.349	1.038
retimed	3.57	1636	932	314	1.451	0.846
2 extra PL	5.00	1884	1077	398	1.791	1.082
3 extra PL	4.55	1822	1120	457	1.791	1.059

## Abbreviations

The following abbreviations are used in this manuscript:

DSO	Digital Storage Oscilloscope
ADC	Analog to Digital Converter
FPGA	Field Programmable Gate Array
ASIC	Application Specific Integrated Circuit
ENOB	Effective Number Of Bits
SFDR	Spurious-Free Dynamic Range
MSB	Most Significant Bit
finFET	fin Field Effect Transistor
DSP	Digital Signal Processing
